# Overweight and obesity of school-age children in El Salvador according to two international systems: a population-based multilevel and spatial analysis

**DOI:** 10.1186/s12889-020-08747-w

**Published:** 2020-05-14

**Authors:** Wilton Pérez, Paul Melgar, Ana Garcés, Ana Daysi de Marquez, Gerardo Merino, Carolina Siu

**Affiliations:** 1 Institute of Nutrition of Central America and Panama-INCAP, Calzada Roosvelt 6-25, zona 11, Guatemala, Guatemala; 2 Consejo Nacional de Seguridad Alimentaria y Nutricional-CONASAN, San Salvador, El Salvador

**Keywords:** El Salvador, IOTF-Cole, Multilevel, Obesity, Overweight, School-aged, Spatial, WHO-2007

## Abstract

**Background:**

The World Health Organization (WHO-2007) and the International Obesity Task Force (IOTF-Cole) systems assess child weight status. However, derived estimations often differ. We aimed to a) compare the prevalence of overweight and obesity, b) analyze individual and contextual factors associated with child weight using multilevel analysis and c) explore the spatial distribution of overweight and obesity using both classification systems.

**Methods:**

We used data from the 2015/2016 National School Height and Weight Census in El Salvador. Information on 111,991 children aged 6.0–9.9 years attending the first grade was analyzed. Body mass index Z-score (BMIZ), overweight and obesity were defined with both classification systems. Weighted kappa was used to measure agreement. Child, school and municipal potential determinants of BMIZ were examined by multilevel analysis. Municipal spatial clustering of overweight and obesity was tested using Moran’s Index and Getis-ord Gi* statistics.

**Results:**

The combined prevalence of overweight and obesity was higher according to the WHO system than the IOTF (30.4% vs 23.1%). The weighted kappa was 0.83. Boys, children attending urban schools, children attending private schools, and children residing in municipalities with high human development index had higher BMIZ than their counterparts. The Moran’s indexes were positives and significant. Clusters of high prevalence (above the national prevalence) of overweight and obesity were found in 29 municipalities using the WHO and IOTF systems. For obesity, 28 and 23 municipalities in clusters of high prevalence were detected using the WHO and IOTF criteria, respectively.

**Conclusions:**

Overweight and obesity is high among school-age children in El Salvador. The prevalence of overweight and obesity was higher when using the WHO system, as compared to the IOTF system. Irrespective of the classification system, the multilevel and spatial analysis derived similar interpretations. These results support the need for national preventive interventions with targeting strategies to reduce overweight and obesity in school-age children.

## Background

The prevalence rate of overweight and obesity (ow/ob) in children and adolescents is an increasing public health problem worldwide [1]. Although data from 1990 to 2015 in most high-income countries shows that ow/ob in children aged 5–17 years is plateauing, in low-and middle-income countries (LMIC) it is rising [1, 2]. The country variations in the obesity prevalence -based on the WHO reference- ranged from 1.2% (Cambodia) to 33% (Nauru) in girls, and in boys from 0.5% (Uganda) to 33% (Cook Islands) in 2015 [1]. The World Health Organization called for a halt to the increasing prevalence of ow/ob in children and adolescents by 2025 [3]. Nonetheless, progress in LMIC is lagging behind.

An age-dependent BMI (weight / square height) is useful to assess their nutritional status as children are growing [4]. In order to classify child weight, the World Health Organization WHO-2007 and International Obesity Task Force IOTF references are two international growth systems often applied [5, 6]. Previous studies, however, reported differences in prevalence estimations regarding the classification system. For instance, studies in Europe, Asia, and Latin America found a higher prevalence of ow/ob using the WHO system when compared with results from the IOTF system [7–9].

National nutrition surveillance systems require criteria to define, analyze determinants and prioritize high-risk places of child obesity. The study of factors associated with weight across individual and contextual levels supports public health interventions as excess weight increases the risk of adverse comorbidities, such as type 2 diabetes [3]. For example, factors such as age and sex and household poverty relate to child ow/ob [10]. Additionally, these surveillance systems apply tools like disease mapping. Although multilevel analysis partly measures variability information explained by the contextual level, this modeling ignores the spatial heterogeneity and correlation in health outcomes across geographical areas [11]. Accounting for spatial dependency makes it possible to determine whether a health outcome (e.g., obesity) is clustered in certain places. In the United Kingdom, for instance, researchers and stakeholders use spatial visualization to address obesity policies [12].

Despite the differences reported between the WHO and IOTF, limited evidence exists about these gaps in LMIC. Thus, we focused our research on whether the WHO and IOTF criteria affect the independent association between child, school and contextual factors and body mass index by using a multilevel approach. Furthermore, it is unclear how the internal geographical distribution of prevalence of ow/ob change by classification criteria. Thus, we compared the number and location of municipalities in high clusters of ow/ob by the classification criteria.

## Methods

### Study setting

**Fig. 1:**
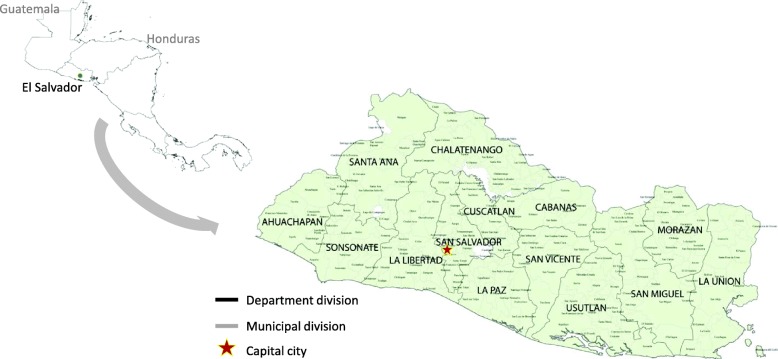
Map of El Salvador, departments and municipalities. The red start on the map indicates the capital city (San Salvador). Maps were built with GeoDa software, version1.8.16.41. Open Source Software Tool (http://geodacenter.github.io). The open source DIVA-GIS repository was used to download each map with its administrative divisions (www.diva-gis.org)

El Salvador is a lower middle-income country located in Central America with a population of 6.3 million inhabitants in 2015 (Fig. 1). The country is administratively divided into 14 regional divisions named departments and 262 municipalities. Each municipality has its own autonomous government responsible for implementing local developmental plans accordingly to national policies. The population size of municipalities varies from 637 to 320,000 inhabitants. At the national level the population is 62% urban. The median by municipality is 34.6% and the range goes from 4.8 to 100%.

Around 28% of the population are children less than fifteen years of age, and 9.8% are aged 6–9 years [13]. One-third of the population lives in poverty and according to the human development index, which ranges from 0 (low development) to 1 (high development), El Salvador ranks as the 116th country with an index of 0.666 [14].

El Salvador has faced a rapid nutrition transition during the last three decades. As a consequence, one-third of males and two-thirds of females more than 20 years old were overweight (BMI ≥ 25) in 2013 [15]. Additionally, in children less than five ow/ob doubled from 1990 to 2013, affecting up to 6 % in 2013 [16].

### Study design and population

This was a secondary data analysis based on a cross-sectional design using the 2015/2016 fourth height and first weight National School Census in El Salvador. The school census has national coverage. All children aged 6.0–9.9 years from public (state funded) and private schools attending the first grade conformed the study sample. Participation rate (based on the 2015-official first-grade enrollment) was 91.1%. Reasons for non-participation may have been either non-attendance on the day of the measurement, or the child’s age was outside of the 6 to 9-year interval. The original dataset has 112,349 records of children aged from six to nine years. Biologically implausible WHO z-scores for weight (below − 6 standard deviation (SD) or above +5SD), height (below -6SD or above +6SD) and BMIZ (below -5SD or above +5SD) were removed (*n* = 340) [17]. We excluded BMIZ (below -5SD or above +5SD) based on IOTF system (*n* = 18). The analytical dataset has 111,991 records. A total of 4622 schools participated, where 86 and 14% were public and private schools, respectively.

The *National Council for Food Security and Nutrition* carried-out the school census in coordination with the Ministry of Education. The census methodology is described elsewhere [18, 19]. In brief, female teachers were trained in anthropometric techniques to measure height and weight. Weight was measured using a portable calibrated digital scale (SECA) to the nearest 0.1 kg. Each child was weighed wearing his or her school uniform and barefoot, removing any type of objects such as bracelets, watches, coins, keys, jackets, sweaters, socks, and belts. A body meter fixed to a vertical surface was used to measure height. Height was measured without shoes, socks, headbands or bows in a standing upright position looking straight ahead in Frankfurt plane. Teachers assured that shoulders, back, buttocks and heels were touching the vertical body meter.

### Measurements

#### Overweight and obesity

The body mass index (BMI) was computed dividing the weight in kilograms by the square of the height in meters. Then, BMI was converted to Z-scores to assess nutritional status. Children were classified as overweight and obese when the BMI for age and sex Z-score (BMIZ) was greater than + 1 and + 2 standard deviations (sd) from the median of the WHO reference, respectively. In contrast, the IOTF system defines overweight and obesity based on centile curves that pass through BMI cut-off points for overweight (25 kg/m^2^) and obesity (30 kg/m^2^) at age 18 years [6].

#### School and municipal variables

The school location was classified as urban or rural. Additionally, the type of primary school was categorized as public or private. At the municipal level, the human development index was considered for modeling. The human development index (HDI), a composite indicator, combines information about education, health and economic variables at the aggregated level, and it was considered as a socioeconomic status predictor at the municipal level. Data on HDI was available only for the year 2009 [17]. The HDI was dichotomized using the median as the cut-off. Because population size varied across municipalities, it was logarithmically transformed and included in the models.

### Data analysis

The data analysis was carried-out in three stages: 1. Prevalence and agreement determination between classification systems, 2. multilevel analysis, and 3. spatial analysis.

#### Prevalence determination and agreement

For the first stage, the prevalence of overweight and obesity was computed using the WHO and IOTF systems. The *who2007.ado* Stata macro was used to determine the BMIZ [20] and *LMSgrowth* software for the IOTF [21]. To assess the agreement between the two systems across the all categories (thin, normal, overweight and obesity), the Fleiss and Cohen weighted kappa was computed with its respective 95% confidence interval [22]. None 0.0–0.20, minimal 0.21–0.39, weak 0.40–0.59, moderate 0.60–0.79, strong 0.80–0.90 and almost perfect above 0.90 kappa values defined the strength of the agreement.

#### Multilevel analysis

Secondly, the association of predictors at student, school and municipal level on BMIZ was assessed using a three-level multilevel linear model. Multilevel modeling accounts for clustering data and allows combining micro-level (i.e., child) and macro-level (i.e., school, municipality) variables [23]. The intraclass correlation coefficient (ICC) at municipal and school level in relation to weight status was computed. Municipal ICC represented the similarity of students within the same municipality (variance municipality/total variance). The school and municipal ICC represented the similarity of students in the same school and municipality. The likelihood ratio test was determined and the fit of performance was assessed between the new model and null model. Significant fit performing modeling was considered good when *p*-value < 0.05.

#### Spatial analysis

Thirdly, Moran’s index was used to determine global spatial autocorrelation. Moran’s index examines whether an outcome behaves similarly between one location (i.e., municipalities) and its surrounding areas. The range of values of Moran’s index is from − 1 to + 1. Positive Moran values indicate a clustering pattern, while negative values show dissimilarities to nearby areas. Values close to zero indicate randomness. Moran’s index was adjusted for variance instability because of small population size in certain municipalities [24]. The Getis-ord Gi* statistics were applied using the raw municipal prevalence of ow/ob to detect local clusters of municipalities with high prevalence and low prevalence. Positive and negative values of Gi* indicate clustering of high (i.e., a municipality with high values surrounded by other municipalities with high values) and low (i.e., municipality with low values surrounded by other municipalities with low values) municipal prevalence of ow/ob, respectively. The municipal prevalence was smoothed with spatial empirical Bayes technique and then used to compare the ow/ob between high and low clusters. This approach allows determination of a smoothed municipal prevalence using neighboring information [25]. The Queen-1 adjacency matrix was used to define neighboring municipalities that share borders. The municipal island Meanguera Del Golfo was excluded from the spatial analysis. A total of 99,999 Monte Carlo replications were simulated for testing hypothesis and significance was assessed when *p*-value < 0.05. McNemar’s test was used to compare differences in the location of municipalities detected in high clusters of ow/ob according to the WHO and IOTF systems. Each municipality was categorized as 1 if it was located in a high cluster of ow/ob and 0 otherwise. All statistical analyses were conducted in Stata 14 using the xtmixed for modeling. Spatial analysis was performed in GeoDa 1.8.16.41.

## Ethical considerations

The data for this analysis were obtained with permission of the Ministry of Health of El Salvador with reference letter No. 2017–6000-26. Dataset is anonymous.

## Results

Of 112,009 school-children records, 111,991 were used for analysis and 51% were boys (Table 1). Around 54% percent were rural and 14% from private schools. Average age was 7.4 years. More than three-quarters were between the ages of 7.0 and 9.0.

**Table 1:** Background information of school-aged children in the analytical dataset, El Salvador, 2015/2016

Characteristics	Boys (***n*** = 57,481)		Girls (***n*** = 54,510)	
	Number	Percentage	Number	Percentage
**Age in years, mean (sd**^**1**^**)**	7.4 (0.59)		7.4 (0.56)	
**Place of residence**				
Urban	26,474	46.0	25,868	47.4
Rural	31,007	53.9	28,642	52.5
**School type**				
Public	49,272	85.7	46,473	85.2
Private	8209	14.2	8037	14.7
**Child age in years**				
6.0 to 6.9	10,516	18.2	11,617	21.3
7.0 to 7.9	38,890	67.6	36,782	67.4
8.0 to 8.9	6478	11.2	5054	9.2
9.0 to 9.9	1597	2.7	1057	1.9
**Anthropometric measurements**				
Height in cm mean (sd)	120.5 (5.88)		119.7 (5.81)	
Height-for-age Z-score mean (sd)	−0.70 (1.04)		−0.63 (1.01)	
Weight in kg mean (sd)	24.7 (5.46)		24.1 (5.39)	
Weight-for-age Z-score mean (sd)	−0.04 (1.33)		−0.02 (1.33)	
BMI kg/m^2^ mean (sd)	16.8 (2.70)		16.7 (2.77)	
WHO Z-score mean (sd)	0.56 (1.41)		0.43 (1.24)	
IOTF Z-score mean (sd)	0.51 (1.18)		0.49 (1.17)	

^1^ sd: standard deviation

Average height was 120.5 cm (sd: 5.88) in boys and 119.7 cm (sd: 5.81) in girls. The mean weight in boys and girls was 24.7 kg (sd: 5.46) and 24.1 (sd: 5.39), respectively. The average BMIZ based on the WHO system was 0.56 for boys (sd: 1.41) and 0.43 for girls (sd: 1.24), while the IOTF system found 0.51 for boys (sd: 1.18) and 0.49 for girls (sd: 1.17).

### Prevalence and agreement

**Fig. 2:**
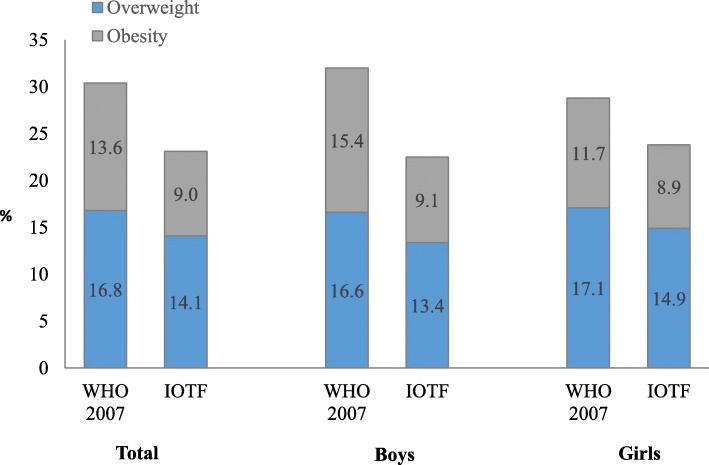
Prevalence of overweight and obesity by child’s sex and classification system, El Salvador, 2015/2016

30.4% (13.6% overweight and 16.8% obesity), 32.0% of boys and 28.8% of girls, were found to be over the recommended healthy weight based on the WHO reference (Fig. 2). On the other hand, the IOTF system reported 23.1% (14.1% overweight and 9.0% obesity), with 22.5% in boys and 23.8% in girls. Regardless of sex and classification system, overweight and obesity was higher in both urban and private schools than those from rural and public schools (Tables 2 and 3). Additionally, ow/ob was higher among younger children in comparison to older ones. Differences in the prevalence of overweight and obesity between the WHO and IOTF system ranged from 4.5 to 10.9%. For obesity alone, these figures varied from 2.2 to 9.3%. These differences were higher in boys than in girls.

**Table 2:** Prevalence of combined overweight and obesity of school-aged children based on the WHO and IOTF growth references, in the analytical dataset, El Salvador, 2015/2016

Characteristic	Prevalence (%) [95%CI]		Difference (%)	Prevalence (%) [95%CI]		Difference (%)
	WHO	IOTF	WHO - IOTF	WHO	IOTF	WHO - IOTF
	Boys	Boys		Girls	Girls	
**Place of residence**						
Urban	38.9 [37.4–40.3]	28.2 [26.7–29.7]	10.7	35.1 [33.8–36.3]	29.5 [28.4–30.6]	5.6
Rural	26.6 [25.5–27.8]	17.6 [16.6–18.6]	9.0	23.6 [22.5–24.7]	18.7 [17.7–19.8]	4.9
**School type**						
Public	29.9 [28.8–31.1]	20.3 [19.3–21.3]	9.6	26.8 [25.7–27.8]	21.7 [20.8–22.7]	5.1
Private	46.4 [45.0–47.8]	35.5 [34.1–37.0]	10.9	42.2 [40.8–43.5]	36.0 [34.6–37.3]	6.2
**Child age in years**						
6.0 to 6.9	35.5 [34.2–36.8]	24.6 [23.4–25.8]	10.9	31.5 [30.1–32.8]	26.2 [25.0–27.5]	5.3
7.0 to 7.9	32.6 [30.9–34.5]	23.0 [21.3–24.8]	9.6	29.6 [27.8–31.4]	24.3 [22.7–26.0]	5.3
8.0 to 8.9	26.6 [24.0–29.4]	17.3 [15.2–19.6]	9.3	21.4 [19.6–23.6]	16.9 [15.3–18.6]	4.5
9.0 to 9.9	24.5 [22.1–27.0]	15.8 [13.7–18.2]	8.7	18.7 [16.3–21.3]	14.1 [11.8–16.9]	4.6

**Table 3:** Prevalence of obesity of school-aged children based on the WHO and IOTF growth references, in the analytical dataset, El Salvador, 2015/2016

Characteristic	Prevalence (%) [95%CI]		Difference	Prevalence (%) [95%CI]		Difference
	WHO	IOTF	WHO - IOTF	WHO	IOTF	WHO - IOTF
	Boys	Boys		Girls	Girls	
**Place of residence**						
Urban	20.3 [19.0–21.6]	12.3 [11.4–13.4]	8.0	15.2 [14.3–16.1]	11.7 [11.0–12.4]	3.5
Rural	11.3 [10.5–12.1]	6.3 [5.7–6.9]	5.0	8.5 [7.9–9.2]	6.3 [5.8–6.9]	2.2
**School type**						
Public	13.5 [12.7–14.3]	7.7 [7.2–8.3]	5.8	10.2 [9.6–10.9]	7.7 [7.2–8.2]	2.5
Private	26.7 [25.5–28.0]	17.4 [16.2–18.5]	9.3	20.1 [19.1–21.2]	15.7 [14.7–16.6]	4.4
**Child age in years**						
6.0 to 6.9	16.9 [15.8–17.9]	10.1 [9.3–10.8]	6.8	12.8 [11.9–13.7]	10.4 [9.6–112.4]	2.4
7.0 to 7.9	15.9 [14.4–17.4]	9.4 [8.4–10.5]	6.5	12.0 [10.9–13.2]	9.0 [8.2–10.0]	3.0
8.0 to 8.9	11.5 [9.9–13.3]	6.1 [5.0–7.4]	5.4	7.9 [6.8–9.2]	5.2 [4.4–6.1]	2.7
9.0 to 9.9	10.8 [9.2–12.5]	5.5 [4.4–6.9]	5.3	5.2 [3.9–7.0]	2.9 [2.0–4.1]	2.3

The overall kappa value was 0.83[95%CI: 0.829–0.834] (strong agreement) between the WHO and the IOTF system (Table 4). No differences were observed in kappa by sex, school location and type of school (Additional file, Table S1). By age, kappa tended to decrease in older ages, mainly after 8 years of age, reporting a substantial concordance.

**Table 4:** Comparing the nutritional status of the school-aged child by categories of the WHO (columns) and IOTF (rows) growth references, El Salvador, 2016. Absolute numbers are shown in each cell

		WHO criteria				
		Thinness (BMIZ ≤ −2)	Normal (2 < BMIZ≤1)	Overweight (1 < BMIZ< 2)	Obese (BMIZ ≥2)	Total
**IOTF criteria**	Thinness (BMI ≤ 18.5 at age 18)	2037	7511	0	0	9566
	Normal (18.5 < BMI ≤ 24.9 at age 18)	0	68,017	8458	0	76,475
	Overweight (24.9 < BMI < 30.0 at age 18)	0	0	10,695	5186	15,881
	Obese (BMI ≥ 30 at age 18)	0	0	0	10,087	10,087
	Total	2037	75,528	19,153	15,273	111,991

### Multilevel analysis

In the random effect part, the partition variance was derived from the null model (no explanatory variables included) and adjusted models (Table 5, Table 6). The ICC at student level for the WHO system explained slightly over 92.9% of the total variance. At the school/municipal and municipal alone, the ICC was 7.03 and 1.6%, respectively. These figures at the student, school/municipal and municipal alone level were 92.7, 5.6 and 1.6% respectively for the IOTF system. Despite the low explained variance at school and municipality, the multilevel model (including all predictors) was significantly preferred over the single model (not accounted for clustering data) and perform better than the null model (no predictors). It means that BMIZ behaved as dependent observations, with some level of clustering at the school and municipal level.

**Table 5:** Multilevel linear regression model of body mass index Z-score using the WHO classification system, in the analytical dataset, El Salvador, 2015/2016

Characteristic	Intercept only model	Student fixed predictors	School predictors	Municipal predictors Full model
	Coefficient (95% CI)	Coefficient (95% CI)	Coefficient (95% CI)	Coefficient (95% CI)
Intercept	0.39 (0.37, 0.42)^a^	1.54 (1.44, 1.65)^a^	1.36 (1.26, 1.46)^a^	1.36 (1.15, 1.56)^a^
**Student level**				
Sex (ref.: girls)		0.15 (0.13, 0.16)^a^	0.14 (0.13, 0.16)^a^	0.14 (0.13,0.16)
Age		−0.16 (−0.17, −0.15)^a^	−0.15 (−0.16, − 0.13)^a^	− 0.15 (− 0.16, − 0.13)^a^
**School level**				
Location (ref.: rural)			0.23 (0.20, 0.26)^a^	0.22 (0.20, 0.25)^a^
Type (ref.: public)			0.33 (0.29, 0.37)^a^	0.32 (0.29, 0.36)^a^
**Municipal level**				
Human development index (ref.: 0.68 or more)				0.12 (0.08, 0.17)^a^
Logarithm of population size				−0.007 (−0.026, 0.012)^b^
**Random effects**				
Student level variance	1.646	1.635	1.636	1.63
School/municipal level variance	0.0959	0.0904	0.0640	0.063
Municipal level variance	0.0285	0.0271	0.0130	0.009
Student ICC	92.9%	93.3%	95.5%	95.4%
School ICC	7.03%	6.7%	4.5%	4.5%
Municipal ICC	1.6%	1.5%	0.7%	0.5%
Deviance	3909.49 (Reference)	886.54	1642.89	1680.45
*P*-value	< 0.001	< 0.001	< 0.001	< 0.001

Ref.: category of reference. Numbers in parentheses represent the 95% confidence interval. *ICC* intra-class correlation coefficient. ^a^*p*-value < 0.01, ^b^*p*-value = 0.478

**Table 6:** Multilevel linear regression model of body mass index Z-score using the IOTF classification system, in the analytical dataset, El Salvador, 2015/2016

Characteristic	Intercept only model	Student fixed predictors	School predictors	Municipal predictors Full model
	Coefficient (95% CI)	Coefficient (95% CI)	Coefficient (95% CI)	Coefficient (95% CI)
Intercept	0.41(0.38, 0.43)^a^	1.51 (1.22, 1.41)^a^	1.36 (1.26, 1.45)^a^	1.36 (1.18, 1.54)^a^
**Student level**				
Sex (ref.: girls)		0.040 (0.027, 0.054)^a^	0.040 (0.026, 0.053)^a^	0.040 (0.026, 0.053)^a^
Age		−0.15 (−0.16, − 0.13)^a^	−0.14 (−0.15, −0.12)^a^	−0.14 (− 0.15, − 0.12)^a^
**School level**				
Location (ref.: rural)			0.20 (0.18, 0.23)^a^	0.20 (0.17, 0.22)^a^
Type (ref.: public)			0.28 (0.25, 0.32)^a^	0.28 (0.25, 0.31)^a^
**Municipal level**				
Human development index (ref.: 0.68 or more)				0.11 (0.070, 0.15)^a^
Logarithm of population size				−0.0068 (−0.024, 0.010)^b^
**Random effects**				
Student level variance	1.290	1.285	1.286	1.286
School/municipal level variance	0.0780	0.0730	0.0526	0.0525
Municipal level variance	0.0225	0.0213	0.0104	0.0076
Student ICC	92.7%	93.1%	95.3%	95.5%
School ICC	5.6%	5.2%	4.6%	4.4%
Municipal ICC	1.6%	1.5%	0.7%	0.5%
Deviance	3999.86 (Reference)	628.44	1351.05	1389.26
*P*-value	0.0000	0.0000	0.0000	0.0000

Ref.: reference category. Numbers in parenthesis represent the 95% confidence interval. *ICC* intra-class correlation coefficient. ^a^*p*-value < 0.01. ^b^*p*-value = 0.443

The fixed effects in both models showed similar conclusions. Boys, children attending urban schools, and children from private schools had higher BMIZ than girls, rural and public school children. The regression coefficients by sex were higher using the WHO system (0.14, *p*-value < 0.01) than the IOTF system (0.04, *p*-value < 0.01). Those municipalities with an HDI above 0.68, showed higher BMIZ (WHO: 0.12, *p*-value < 0.01 vs IOTF: 0.10, *p*-value < 0.01) than those with a HDI less than 0.68.

### Spatial analysis

**Fig. 3:**
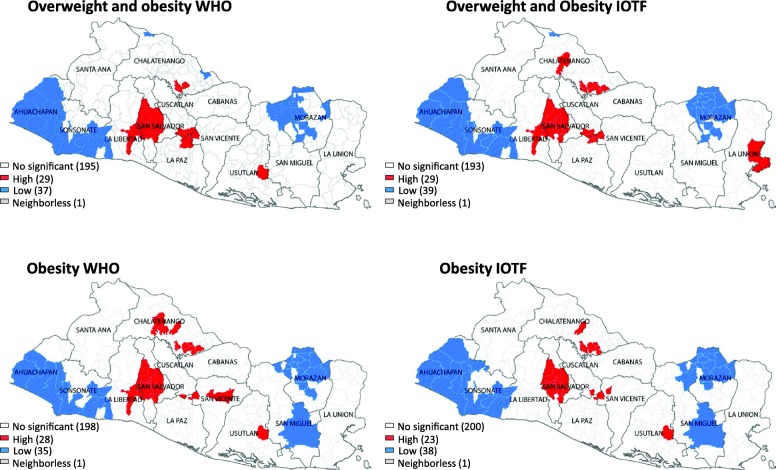
Map of spatial clusters of overweight and obesity using the WHO-2007 and the IOTF systems, El Salvador, 2015/2016. Maps were built with GeoDa software, version1.8.16.41. Open Source Software Tool (http://geodacenter.github.io). The open source DIVA-GIS repository was used to download the map with its administrative divisions (www.diva-gis.org)

We examined the prevalence of ow/ob across the municipalities using the WHO and IOTF systems. The Moran’s Index for the combined overweight and obesity and obesity alone were 0.434 and 0.359, respectively (*p*-value = 0.001) using the WHO system. These figures using the IOTF system were 0.561 and 0.552 (*p*-value = 0.001). Then, the local clustering based on Getis-Ord Gi* identified significant municipal clusters for overweight and obesity (Fig. 3).

In the WHO and IOTF system, 29 municipalities in clusters of high combined ow/ob were detected, and 24 were common to both systems (exact McNemar’s test, *p*-value = 0.99). On the other hand, for obesity, in the WHO criteria, five more municipalities in high clusters were found compared with the IOTF system (28 vs 23), with 20 municipalities overlapping between both systems (exact McNemar’s test, *p*-value = 0.22). In a supplementary file, the names of municipalities located in clusters of high prevalence of ow/ob are provided (Additional file, Table S2).

The average smoothed prevalence of combined ow/ob was 37% in high clusters and 21% in low clusters using the WHO system. For obesity, high clusters depicted a prevalence of 18 and 8% for low clusters. Based on the IOTF system the smoothed prevalence of ow/ob in high clusters was 30 and 15% in low clusters. The average smoothed prevalence of obesity was 13% in high clusters and 5% in low clusters.

## Discussion

This unique population-based study estimated overweight and obesity in a large sample of school-age children from El Salvador with two international classification systems. Our findings confirmed that the WHO and the IOTF criteria resulted in different estimations of overweight and obesity. Although the multilevel analysis provided information of factors associated to weight, the explained variation in the municipal level was low. The spatial analysis informed about municipal correlations of overweight and obesity within the country undetected by multilevel modeling. This study demonstrated that despite the distinction between both classification systems, the interpretations derived by applying multilevel and spatial approaches were similar.

We found prevalence of ow/ob to be higher using the WHO system than the IOTF criteria. This finding was consistent with previous studies in the literature [7–9]. The nature of the referent population from both systems might explain these differences. For instance, the WHO-2007 system is a smoothed growth curve transition built from two data sources: first, the WHO-2006 growth standard (for children < 5 years) and second, the 1977 National Center for Health Statistics (NCHS/WHO) growth charts. The NCHS/WHO growth reference is constituted from a non-obese sample of children aged 1–24 years collected from 1963 to1974. Thus, the referent population might represent a healthier group and therefore be more sensitive to diagnoses of excess weight. Conversely, the IOTF was developed combining more recent BMI data of children aged 2–18 years from six national representative surveys from 1963 to 1993. Thus, the shift towards an increased BMI may underestimate ow/ob using the IOTF criteria in our study sample. From 6 to 10 years, the IOTF cut-points to define overweight are 1.1 BMI units greater than the WHO cut-points for boys and 0.6 for girls [5, 6]. For obesity, the mean IOTF and WHO cut-points differ 1.9 and 1.0 BMI units for boys and girls, respectively. The former explains the higher differences of obesity prevalence between the WHO and IOTF systems in boys as compared to girls. These methodological discrepancies might unfold why the WHO criteria is a more sensitive screening system for ow/ob, and derives higher prevalence estimates when compared with the IOTF. The BMIZ differences between boys and girls were smaller using the IOTF system than WHO system. This is due to similarities of BMIZ values by sex obtained with the IOTF as compared to the WHO system. For instance, the cut-point gap of BMIZ for overweight between a boy and girl at 9 years is 0.03 and 0.4 using the IOTF and WHO systems respectively [5, 6].

The multilevel analysis showed comparable associations and variations using both systems. The fixed associations found were in line with other studies in Latin American countries [26, 27]. In addition, we found a lower explained variation of BMIZ attributable to schools and municipalities, as compared with children in both systems. It was consistent with findings that reported low BMI variation in school-age children and adults at cluster level in low-income settings [28, 29]. This finding is relevant when targeting interventions. For instance, in Honduras, Morris et al. pointed out that school estimations on linear growth retardation (i.e., low height-for-age) were unreliable for targeting interventions at the school level [19]. However, municipal estimations were robust enough to be used for that purpose. Hence, an implication from the findings of our study is that because of the low explained BMIZ variation at school and municipal level, both systems support the implementation of preventive interventions at the national level [29].

Older children had lower BMI Z-scores and therefore showed a lower prevalence of overweight and obesity than younger children. A suggested explanation is grade retention. Children experiencing grade repetition may depict different social and nutritional backgrounds. For instance, in Brazil low socioeconomic status was associated with grade retention in school children [30].

The BMIZ was higher in children attending urban and private schools, and in school children from municipalities depicting an HDI above the median. In LMIC, socioeconomically advantaged children are more likely to consume high-density energy food products and sweetened beverages [31]. A national study in school-age children from Mexico showed a direct association between high socioeconomic status and unhealthy dietary patterns [32]. Generally, these unhealthy diets are constituted of foods and beverages with high content of sodium, added sugar and fats, and are associated with increasing obesity and cardiometabolic risk factors [33].

We identified a group of municipalities with high and low ow/ob through spatial clustering analysis. There were no significant differences in the number of high-prevalence municipalities detected by both systems. The highest prevalence of ow/ob –and obesity alone– clustered in municipalities from the central, northern and southern parts of El Salvador. This analysis suggests the existence of contextual factors that might explain spatial clustering. For instance, municipal clusters of high prevalence of ow/ob -and obesity alone- were located in municipalities with higher HDI as compared with clustering of low ow/ob (data not shown). In Delhi, India, families with higher income were living in high fast food restaurant density areas [34]. In contrast, a study conducted in Sao Paulo, Brazil, found an opposite relationship [35]. Further studies are needed to analyze the association between contextual factors and clusters of high prevalence of overweight and obesity. A small proportion of the total variance in BMIZ was at the municipal level which indicates that the spatial context has little importance in explaining the individual differences in the body weight of child. However, ICC does not account for spatial dependency in the body weight among nearby municipalities. Thus, the application of spatial measurements such as Moran’s index and Getis-Ord Gi* statistics addresses the spatial correlation of a health outcome, indicating that municipalities in the same vicinity depict more similar prevalence of overweight and obesity than municipalities that are far apart. Therefore, regardless of the classification system, the spatial lens provides avenues to program design managers to localize municipalities with high prevalence of ow/ob and target interventions.

The findings of our study should be interpreted keeping in mind the following limitations that affected our data collection. First, child weight was measured using the regular clothes, and adjustments for this extra weight were not performed during data collection. It might bias the magnitude of prevalence, but it is unlikely that it affects the associations and spatial variations obtained in our study. Reliability and accuracy data for anthropometric measurements was not available, but biologically implausible values as recommended by WHO were very low in the database. Standard deviation for height-for-age, weight-for-age and BMIZ were in the recommended range of good quality [36]. The percentage of terminal digit preference in height was almost 50% for measurements with the decimals 0 and 5, which may be explained by the use of a tape measure. However, the bias on the terminal digit preference in prevalence and Z-scores in older children has minimal effect on prevalence estimations [37]. Despite the limitations, it is worth noting that our findings were consistent with results reported from previous studies at the international level. School attendance at the moment of the interview was more than 90%, which assures precision in the estimations and reduced potential selection bias.

## Conclusions

In this study, the comparison between two international classification systems of child weight using multilevel and spatial approaches provided similar interpretations. The child weight variability was strongly accounted for individual factors and weakly by municipal factors. Child characteristics, and socioeconomic school and municipal factors were independently associated with child weight. The prevalence of overweight and obesity was dependent on space and a number of municipalities were found to have a higher prevalence of overweight and obesity (above the national prevalence) using the WHO and IOTF criteria. Future research is required to analyze other potential individual (e.g, behaviors), family (e.g., poverty), and environmental factors (e.g., food, built and social environment) related with child weight. These results support the need to prioritize national preventive interventions with targeting strategies to reduce overweight and obesity.

## Supplementary information

**Additional file 1: Table S1.** Level of agreement of nutritional status of the school-aged child by sex, age, type of school and residence, El Salvador, 2015/2016

**Additional file 2: Table S2.** Municipalities in high clusters of overweight and obesity based on the WHO and IOTF, El Salvador, 2015/2016. The bold names represent the larger geographical division (departments) of the country.

## Data Availability

The datasets used and/or analyzed during the current study are not publicly available because they belong to the government of El Salvador. However, the datasets may be available from the author (ADM: daysi.demarquez@gmail.com) on reasonable request.

## Abbreviations

LMIC: Low- and middle- income countries
BMIZ: Body mass index Z-score
HDI: Human development index
ICC: Intra-class correlation
OW/OB: Overweight and obesity
WHO: World Health Organization
IOTF: International Obesity Task Force
