# Supplementary material for: Overweight and obesity of school-age children in El Salvador according to two international systems: a population-based multilevel and spatial analysis
Source: BMC Public Health. 2020 May 14;20:687. doi: 10.1186/s12889-020-08747-w (PMC7227092; doi:10.1186/s12889-020-08747-w)
Supplement: Supplementary file 1 — Additional file 1: Table S1. Level of agreement of nutritional status of the school-aged child by sex, age, type of school and residence, El Salvador, 2015/2016 [file 12889_2020_8747_MOESM1_ESM.docx]

**Additional file 1**

Table S1 Level of agreement of nutritional status of the school-aged child by sex, age, type of school and residence, El Salvador, 2015/2016

| **Characteristic** | **Quadratic weighted**  **kappa** | **95% confidence interval** |
| --- | --- | --- |
| **Sex** |  |  |
| Boys | 0.813 | 0.810-0.815 |
| Girls | 0.853 | 0.852-0.854 |
|  |  |  |
| **Child age in years** |  |  |
| 6.0 to 6.9 | 0.841 | 0.837-0.842 |
| 7.0 to 7.9 | 0.834 | 0.833-0.836 |
| 8.0 to 8.9 | 0.792 | 0.786-0.799 |
| 9.0 to 9.9 | 0.782 | 0.763-0.791 |
|  |  |  |
| **Place of residence** |  |  |
| Rural | 0.806 | 0.804-0.809 |
| Urban | 0.847 | 0.846-0.850 |
|  |  |  |
| **School type** |  |  |
| Public | 0.821 | 0.819-0.821 |
| Private | 0.862 | 0.861-0.862 |
