# Supplementary material for: Overweight and obesity of school-age children in El Salvador according to two international systems: a population-based multilevel and spatial analysis
Source: BMC Public Health. 2020 May 14;20:687. doi: 10.1186/s12889-020-08747-w (PMC7227092; doi:10.1186/s12889-020-08747-w)
Supplement: Supplementary file 2 — Additional file 2: Table S2. Municipalities in high clusters of overweight and obesity based on the WHO and IOTF, El Salvador, 2015/2016. The bold names represent the larger geographical division (departments) of the country. [file 12889_2020_8747_MOESM2_ESM.docx]

**Additional file 2**

Table S2 Municipalities in high clusters of overweight and obesity based on the WHO and IOTF, El Salvador, 2015/2016. The bold names represent the larger geographical division (departments) of the country.

| High clusters for combined overweight and obesity, WHO system |
| --- |
| **Chalatenango:** Azacualpa, San Francisco Lempa, San Luis del Carmen, San Miguel de Mercedes. **Cuscatlán:** Candelaria, San Cristobal, San Ramón, Santa Cruz Analquito. **La Libertad:** Antiguo Cuscatlán, Nueva San Salvador. **La Paz:** Jerusalén, Mercedes de la Ceiba, Paraíso de Osorio, San Pedro Nonualco, Santa Maria Ostuma. **San Salvador:** Apopa, Ayutuxtepeque, Cuscatancingo, Ilopango, Mejicanos, Nejapa, San Marcos, San Salvador, Santo Tomas, Soyapango, Tonacatepeque, Delgado. **San Vicente:** Verapaz. **Usulután:** Santa Elena |
| High clusters for obesity, WHO system |
| **Chalatenango:** Azacualpa, El Paraiso, Potonico, San Antonio los Ranchos, San Francisco Lempa, San José Cancasque, San Miguel de Mercedes, Santa Rita, Tejutla. **Cuscatlan:** Santa Cruz Analquito. **La Libertad:** Antiguo Cuscatlan, Nueva San Salvador. **La Paz** Mercedes de la Ceiba. **San Salvador:** Apopa, Ayutuxtepeque, Cuscatancingo, Ilopango, Mejicanos, Nejapa, San Marcos, San Salvador, Santo Tomás, Soyapango, Tonacatepeque, Delgado. **San Vicente:** Apastepeque, Verapaz. **Usulután:** Santa Elena |
| High clusters for combined overweight and obesity, IOTF system |
| **Chalatenango:** Azacualpa, El Paraíso, Potonico, San Antonio los Ranchos, San Francisco Lempa, San José Cancasque, San Miguel de Mercedes, Santa Rita, Tejutla. **Cuscatlán:** Santa Cruz Analquito. **La Libertad:** Antiguo Cuscatlán, Nueva San Salvador. **La Paz:** Mercedes de la Ceiba. **San Salvador:** Apopa, Ayutuxtepeque, Cuscatancingo, Ilopango, Mejicanos, Nejapa, San Marcos, San Salvador, Santo Tomas, Soyapango, Tonacatepeque, Delgado. **San Vicente:** Apastepeque, Verapaz. **Usulután:** Santa Elena |
| High clusters for obesity, IOTF system |
| **Chalatenango:** Azacualpa, Potonico, San Antonio los Ranchos, San Francisco Lempa, San Luis del Carmen, San Miguel De Mercedes, Santa Rita. **San Salvador:** Apopa, Ayutuxtepeque, Cuscatancingo, Ilopango, Mejicanos, Nejapa, San Salvador, Santo Tomas, Soyapango, Tonacatepeque, Delgado. **Cuscatlán:** San Ramón. **La Paz:** Mercedes de la Ceiba. **San Vicente:** San Lorenzo, Verapaz. **Usulután:** Santa Elena |
